# A pilot study of exercise in men with prostate cancer receiving androgen deprivation therapy

**DOI:** 10.1186/1471-2407-12-103

**Published:** 2012-03-21

**Authors:** C Ellen Lee, William D Leslie, YK James Lau

**Affiliations:** 1 Department of Physical Therapy, University of Manitoba, R106 - 771 McDermot Ave., Winnipeg MB R3E 0T6, Canada; 2 Department of Medicine, University of Manitoba, St. Boniface Hospital, 409 Tache Ave., Winnipeg MB R2H 2A6, Canada; 3 Eli Lilly Corporation, Lilly Corporate Center, Indianapolis, Indiana 46285, USA

**Keywords:** Prostate cancer, Androgen deprivation therapy, Walking, Home-based exercise, Bone health, Physical function, Quality of life

## Abstract

**Background:**

Androgen deprivation therapy (ADT) is the mainstay therapy for men with prostate cancer. However, there are musculoskeletal side effects from ADT that increase the risk for osteoporosis and fracture, and can compromise the quality of life of these individuals. The objectives of this study are to determine the efficacy of a home-based walking exercise program in promoting bone health, physical function and quality of life in men with prostate cancer receiving ADT.

**Methods/Design:**

A 12-month prospective, single-blinded, randomized controlled trial will be conducted to compare the Exercise Group with the Control Group. Sixty men with prostate cancer who will be starting ADT will be recruited and randomly assigned to one of the two groups: the Exercise Group will receive instructions in setting up an individualized 12-month home-based walking exercise program, while the Control Group will receive standard medical advice from the attending physician. A number of outcome measures will be used to assess bone health, physical function, and health-related quality of life. At baseline and 12 months, bone health will be assessed using dual-energy X-ray absorptiometry. At baseline and every 3 months up to 12 months, physical function will be evaluated using the Functional Assessment of Chronic Illness Therapy - Fatigue Scale, Activities-specific Balance Confidence Scale, Short Physical Performance Battery, and Six-Minute Walk Test; and health-related quality of life will be assessed using the Functional Assessment of Cancer Therapy Prostate Module and the Medical Outcomes Study 12-item Short Form Health Survey Version 2. A mixed multiple analysis of variance will be used to analyze the data.

**Discussion:**

Musculoskeletal health management remains a challenge in men with prostate cancer receiving ADT. This study addresses this issue by designing a simple and accessible home-based walking exercise program that will potentially have significant impact on reducing the risk of fracture, promoting physical function, and ultimately improving the health-related quality of life in men with prostate cancer receiving ADT.

**Trial registration:**

ClinicalTrials.gov: NCT00834392.

## Background

Prostate cancer is the most commonly diagnosed cancer among men in North America, excluding non-melanoma skin cancer. There are currently 25,500 men in Canada [1] and over 2.3 million men in the United States who have prostate cancer [2]. Despite apparently localized and hence curable disease at presentation, more than one-third of men with prostate cancer will develop recurrent disease. Androgen deprivation therapy (ADT), accomplished by bilateral orchiectomy or the use of a luteinizing hormone-releasing hormone (LHRH) agonist, is the mainstay therapy for recurrent and/or metastatic prostate cancer. The duration of response to ADT using an LHRH agonist ranges from 12 to 18 months, with 20% of patients having a complete prostate specific antigen (PSA) response at 5 years [3]. However, there are a number of side effects of ADT that negatively impact the health-related quality of life in these men. The side effects of such treatment include osteoporosis, [4,5] increased fracture risk, [6-8] poorer quality of life with decline in physical function, [9,10] fatigue, decline in muscle mass, increased fat mass, weight gain, [11,12] reduced psychosocial and cognitive functions [13,14]. Osteoporosis and physical function decline are of particular concern as these are factors that are closely related to risks of falls and fractures in older men [15-18].

Exercise is an easily accessible lifestyle intervention that may have significant positive impact on the quality of life in men with prostate cancer. A number of studies have already shown that exercise improves the quality of life and even survivorship in women with breast cancer [19,20]. However, there are few studies investigating the effects of exercise on bone health and physical function in men with prostate cancer. Walking has been shown to have beneficial effects on bone metabolism in post-menopausal women [21]. We speculate that walking may have a similar effect on bone metabolism in men with prostate cancer receiving ADT. In addition, a study showed that short-term supervised, rigorous resistance exercise could reduce fatigue and improve muscular fitness, and improve some aspects of quality of life in men with prostate cancer receiving ADT [22]. Therefore, it is imperative to explore other forms of exercise that can achieve similar results as resistance exercise, but are more accessible and can be readily incorporated into the long-term lifestyle of men with prostate cancer. The purpose of this study is to determine the efficacy of a home-based walking exercise program in promoting bone health, physical function and quality of life in men with prostate cancer receiving ADT.

## Methods/Design

A 12-month prospective, single-blinded, randomized controlled pilot study will be conducted to compare an Exercise Group with a Control Group. The study will be conducted in the Department of Physical Therapy in the Faculty of Medicine at the University of Manitoba. Patient recruitment will be conducted through Cancer Care Manitoba, with baseline and follow-up sessions at the Department of Physical Therapy at University of Manitoba. The University of Manitoba Research Ethics Board has approved the study protocol (Ethics Reference # H2008:035), which is also in compliance with the Helsinki Declaration.

### Participants

#### Inclusion criteria

Patients will be included in the study if they are men aged 50 years or older, diagnosed with adenocarcinoma prostate cancer, and who will initiate and receive continuous ADT (LHRH agonist alone or in combination with another anti-androgen) for at least 12 months after recruitment. Patients will be required to provide written informed consent to participate in the study.

#### Exclusion criteria

Patients will be excluded from the study if they have severe cardiac disease (New York Heart Association class III or greater), angina, pre-existing osteoporosis with T-score at or below -2.5, stable bone lesion, uncontrolled hypertension (blood pressure > 160/95 mm Hg), moderate to severe aortic stenosis, acute illness or fever, uncontrolled atrial or ventricular dysrhythmias, uncontrolled sinus tachycardia (> 120 beats per minute), third-degree atrio-ventricular heart block, active pericarditis or myocarditis, recent pulmonary embolism, deep vein thrombosis, uncontrolled diabetes, uncontrolled pain, cognitive impairment, history of falls due to balance impairment or loss of consciousness, severe neuromusculoskeletal conditions that limit their ability to perform walking exercise (including ataxia, peripheral or sensory neuropathy, unstable bone lesion, severe arthritis, pathological lower limb fractures within 6 months, lower limb amputation).

#### Recruitment

A total of 60 patients will be consecutively recruited during the initial 3-months of the study from office visits at Cancer Care Manitoba. The attending physician will screen the patients for inclusion criteria (men aged 50 or older), diagnosed with prostate adenocarcinoma, will initiate and receive continuous ADT (LHRH alone or in combination with another anti-androgen for at least 12 months). If the patients meet the preliminary inclusion criteria, the physician will refer them to a clinical research nurse who will confirm that all inclusion and exclusion criteria are met, explain the study protocol, provide information sheet, and obtain written informed consent from any patients who would like to participate in the study. The clinical research nurse will provide a graduate research assistant (GRA1) with the signed consent forms and contact information of the patients who have agreed to participate in the study.

### Assessment sessions

Each participant will attend a total of five assessment sessions: one baseline, and four follow-up sessions at 3, 6, 9, and 12 months after ADT initiation. The Exercise Group will complete telephone survey between follow-up sessions.

### Baseline assessment session

GRA1 will contact the participants to schedule a baseline assessment. GRA1 will collect the socio-demographic and clinical characteristics, followed by primary outcome (HRQOL and physical function) and secondary outcome variables (physical fitness, psychosocial-cognitive functions, and physical activity).

GRA2 (another graduate research assistant) will provide each participant with a pedometer to monitor his ambulatory activities. The participants will be instructed to wear the pedometer during waking hours, except while bathing or swimming. The pedometer will be worn on a waistband, at or slightly anterior to the mid-axillary line. The exact location will be determined by a 50-step trial walk, and the placement that yields the least percentage error in step counts will be used for the individual's daily application. Pedometer daily logs, self-addressed envelopes, and extra batteries will be provided to all participants. At the end of each day, participants will record the date, total daily time wearing the pedometer, and the number of daily steps taken, and then reset the pedometer to zero. Every four weeks, GRA2, will call the participants to remind them to return their pedometer daily logs by mail in the self-addressed envelopes.

#### Randomization of group assignment

GRA1 will be blinded to the group assignments of the participants. Therefore, after the baseline assessment and pedometer instruction, GRA2 will be responsible for the randomized group assignment. GRA2 will assign the participant the first available number from a randomly generated list consisting of "1" and "2". Number "1" is designated as the Exercise Group that will receive instructions in setting up an individualized 12-month home-based walking exercise program in addition to their daily activities. Number "2" is designated as the Control Group that will be advised to perform daily activities as usual and follow the standard medical advice from their attending physician.

#### Exercise group protocol

GRA2 will assess the baseline health and fitness of the participants of the exercise group by using an exercise screening form that includes the revised Physical Activity Readiness Questionnaire (PAR-Q), [23] and other screen questions for exercise contraindications and precautions. If any exercise contraindication is identified (e.g. shortness of breath, severe headache, sudden onset of numbness or weakness), the participant will be excluded from the study and referred to their family physician for investigation. If any exercise precautions is identified (e.g. fever, severe cachexia, extreme fatigue, sickness or bone pain), high-intensity or high-impact exercise will be initially avoided. If severe nausea or calf pain is present, the participants will be advised to consult their family physician for further investigation and clearance prior to beginning the exercise program.

The Exercise Group will follow a walking exercise program that comprises a progressive structured exercise protocol and progressive target daily step counts. The structured exercise protocol will initially begin with a 10-minute walking session, 3 sessions per week. The Exercise Group participants will be instructed to perform a 5-minute warm-up period by walking slowly prior to the 10-minute walking session. There will also be a 5-minute cool-down period by walking slowly, followed by a stretching routine after the 10-minute walking session. The stretching instruction sheet will be provided to the participants. GRA2 will also educate and demonstrate to the participants how the stretches are to be done. During the warm-up/cool-down periods, the participants will maintain an exertion level between 7.5 and 8 on the Borg Scale of Ratings of Perceived Exertion (RPE) [24] (i.e., extremely light level of exertion). During the 10-minute walking session, the participants will gradually increase the walking speeds until they attain and maintain an RPE level between 9 and 13 (i.e., very light to light exertion) according to their baseline physical activity level (sedentary, moderately active, or active) as reported on their self-reported physical activity questionnaire. Sedentary individuals will walk at RPE level 9 (very light exertion), moderately active individuals will walk at RPE level 11 (light exertion), and active individuals will exercise at RPE level 13 (somewhat hard exertion). Each participant's exercise level will be progressed every two week, first in frequency (1-day increment), then duration (5-minute increment), and lastly RPE level. Based on the set progression protocol, the Exercise Group participants will be performing five walking exercise sessions per week, 30 minutes walking per session, and will attain RPE level 15 (heavy exertion) during the walking session by around 18 to 20 weeks. This level of exercise protocol will be maintained for the rest of the study period. The participants are instructed to record the date, start time, exercise duration and maximum RPE attained during each session in an exercise daily log. The participants will also be encouraged to walk at their chosen pace at times outside their structured exercise protocol so as to achieve their assigned target daily step counts. The initial target will be set at 5,000 steps for sedentary individuals, 6000 steps for moderately active individuals, and 7,000 steps for active individuals. The target daily step counts will be progressed every two weeks (1000-steps increment) up to a maximum daily target of 10,000 steps. The participants will be counseled on the warning symptoms of impending cardiovascular events including chest pain or discomfort, shortness of breath, dizziness, severe headache, sudden onset of numbness or weakness, or calf pain suggestive of deep vein thrombosis. They will be strongly advised to discontinue the exercise program and report these symptoms to their attending physician and GRA2 immediately. An exercise advice and instructions sheet will be provided to the Exercise Group participants.

### Between follow-up sessions

For the first 20 weeks, GRA2 will call the Exercise Group participants every two weeks to monitor their safety, adherence, and progress using a short, structured phone survey. The results of the phone survey will be used to guide the adjustments/progression of the structured exercise and step counts protocols. After 20 weeks, GRA2 will conduct the phone survey every four weeks. In addition, the participants will return their exercise daily logs every four weeks.

#### Follow-up sessions

GRA1 will schedule follow-up sessions for all participants at 3, 6, 9, and 12 months after ADT initiation. Primary outcome (HRQOL and physical function) and secondary outcome variables (physical fitness, psychosocial-cognitive functions, and physical activity) will be re-assessed during the follow-up sessions.

#### Data collection

At baseline session, GRA1 will collect independent variables of socio-demographic, clinical, and behavioral factors using a structured assessment questionnaire. Socio-demographic factors will include (1) age, (2) marital status, (3) ethnicity, (4) living arrangement, (5) education, (6) work status, (7) alcohol consumption history, and (8) smoking history. Clinical factors will include (9) current cancer stage, (10) time since diagnosis, (11) treatment intent (i.e., curative vs. palliative). Other baseline data including testosterone level, Gleason score, and comorbidities will be obtained from the medical records.

At baseline and 12-months after ADT initiation, GRA1 will obtain bone mineral density (for *bone health *as part of the primary outcomes) of the lumbar spine, hip and whole body and body composition (for *physical fitness *as part of the secondary outcomes) of the whole body from the dual-energy x-ray absorptiometry (DXA),^a ^and PSA level from the medical records.

At baseline and all follow-up sessions (3, 6, 9, and 12 months), the rest of the primary (HRQOL and physical function) and secondary outcome variables (physical fitness, psychosocial-cognitive functions, and physical activity) will be assessed. *HRQOL *will be assessed using (1) the Functional Assessment of Cancer Therapy Prostate Module (FACT-P) [25] as a disease-specific instrument, and (2) the Medical Outcomes Study 36-item Short Form Health Survey Version 2 (SF-36v2) [26] as generic instrument that is chosen for its depth of coverage, breadth of domains, and widespread use in medical outcomes assessment. *Physical function *will be assessed using (3) the Functional Assessment of Chronic Illness Therapy (FACIT) - Fatigue scale [27] that evaluates self-reported fatigue level; (4) the Activities-specific Balance Confidence (ABC) Scale [28] that evaluates the self-reported confidence in activities of daily living; (5) the Short Physical Performance Battery (SPPB) [29,30] that objectively assesses lower extremity function including standing balance, gait speed, and ability to rise from a chair; and (6) the 6-Minute Walk Test (6MWT) [31] that evaluates walking endurance. *Physical fitness *will be assessed using (7) the body mass index based on the participants' height and weight measurement obtained by a standard medical weight scale. *Psychosocial-cognitive functions *will be assessed using (8) the Multidimensional Health Locus of Control (MHLC) Form C [32] for individuals' beliefs regarding where the control over their health lies; (9) the Center for Epidemiological Studies Depression (CES-D) [33] for depression; and (10) the Prospective Retrospective Memory Questionnaire (PRMQ) for short and long-term memory. *Physical activity *will be assessed using (11) the Rapid Assessment of Physical Activity [34]. *Physical activity *is also assessed based on the completed pedometer daily logs returned by all participants every four weeks.

#### Exercise group

*Exercise adherence *of the Exercise Group participants will be monitored by the exercise daily logs returned every four weeks, and phone surveys conducted by GRA2 every two weeks for the first 20 weeks and every four weeks for the rest of the study period. The phone surveys also monitor *exercise safety and progress*.

### Statistical analysis

#### Primary outcome variables

A mixed multiple analysis of variance (MANOVA) (Group X Time) will be conducted to determine if there is a significant interaction between the between- group (Group) and within-group (Time) comparisons for:

*Bone health *(bone mineral density) at baseline and 12-month follow-up session;

*HRQOL *(FACT-P and SF-36), and *physical function *(FACT-Fatigue, ABC, SPPB, and 6MWT) at baseline, 3, 6, 9, and 12-month follow-up sessions.

### Secondary outcome variables

A mixed MANOVA (Group X Time) will be conducted to determine if there is a significant interaction between the between-group (Group) and within-group (Time) comparisons for:

- *Physical fitness *(body composition) reflected in lean body mass at baseline and 12-month follow-up session;

*Physical fitness *(body mass index), *psychosocial-cognitive functions *(MHLC Form C, CES-D, and PRMQ), and *physical activity *(RAPA, daily step counts based on the pedometer daily logs) at baseline, 3, 6, 9, and 12-month follow-up sessions.

For the Exercise Group, Spearman-rank or Pearson product-moment correlation will be performed to determine if socio-demographic (age, marital status, living arrangement, education, and work status) and clinical factors (current cancer stage, time since diagnosis, treatment intent, testosterone level, Gleason score, comorbidity index, and PSA level) are associated with *exercise adherence *(average percentage of adherence to the target exercise frequency, duration and intensity based on the exercise daily logs) and *exercise safety *(occurrence rate of safety issues from the phone survey).

## Discussion

Bone health management remains a challenge in men with prostate cancer receiving ADT. The primary objective of the study is to determine the feasibility and efficacy of a simple and accessible home-based walking exercise program in promoting bone health, health-related quality of life, and physical function in men with prostate cancer receiving ADT as compared to a control group. This study is significant because a successful home-based exercise program for men with prostate cancer would be cost-effective, and has a potentially significant impact on a number of comorbidities that are related to ADT. This multidisciplinary study lays the foundation for the development of a prevention and wellness program within a multidisciplinary, comprehensive cancer care program for men with prostate cancer.

## Endnote

^a^DXA is part of a standard patient care for men with prostate cancer on ADT. The test is performed to monitor bone mineral density of total body, at lumbar spine and femur sites. Total body composition is based on additional analysis of the total body DXA results.

## Competing interests

All authors do not have any financial or non-financial competing interests to declare in relation to this manuscript. The organizations that the authors were and are currently employed under do not in any way gain or lose financially from the publication of this manuscript. YKJL was involved in this study prior to employment at Eli Lilly Corporation.

## Authors' contributions

CEL conceived the study, participated in the design of the study, coordinated acquisition of data, participated in data analysis and interpretation, drafted and revised critically the manuscript, and also provided final approval for the published version. WDL provided substantial support in the acquisition, analysis and interpretation of bone mineral density data, and also critically reviewed the manuscript and provided final approval for the published version. YKJL conceived the concept and design of the study, provided substantial support in the recruitment of patients, participated in data analysis and interpretation, drafted and revised critically the manuscript, and also provided final approval for the published version. All authors read and approved the final manuscript.

## Pre-publication history

The pre-publication history for this paper can be accessed here:

http://www.biomedcentral.com/1471-2407/12/103/prepub
